# Middle East respiratory syndrome: pathogenesis and therapeutic developments

**DOI:** 10.2217/fvl-2018-0201

**Published:** 2019-04-18

**Authors:** Hani Choudhry, Muhammed A Bakhrebah, Wesam H Abdulaal, Mazin A Zamzami, Othman A Baothman, Mohammed A Hassan, Mustafa Zeyadi, Nawal Helmi, Faisal Alzahrani, Ashraf Ali, Mohammad Khalid Zakaria, Mohammad Azhar Kamal, Mohiuddin Khan Warsi, Firoz Ahmed, Mahmood Rasool, Mohammad Sarwar Jamal

**Affiliations:** 1 Department of Biochemistry, Cancer Metabolism & Epigenetic Unit, Faculty of Science, Cancer & Mutagenesis Unit, King Fahd Medical Research Center, King Abdulaziz University, Jeddah 21589, Saudi Arabia; 2 Life Science & environment Research Institute, National Center for Genome Technology, King Abdulaziz City for Science and Technology (KACST), Riyadh 12371, Saudi Arabia; 3 Department of Basic Medical Sciences, College of Medicine & Health Sciences, Hadhramout University, Yemen; 4 Hematology Lab Unit, King Fahd Medical Research Center, King Abdulaziz University, Jeddah 21589, Saudi Arabia; 5 Department of Medical Laboratory Technology, Faculty of Applied Medical Sciences, King Abdulaziz University, Jeddah 21589, Saudi Arabia; 6 Department of Science of Agriculture, Food and Environment (SAFE), University of Foggia, Via Napoli, 25 – 71122, Foggia, Italy; 7 The Pirbright Institute, Ash Road, GU240NF, Surrey, United Kingdom; 8 Department of Biochemistry, University of Jeddah, Jeddah 23890, Saudi Arabia; 9 Center of Excellence in Genomic Medicine Research, King Abdulaziz University, Jeddah 21589, Saudi Arabia

**Keywords:** Arabian Peninsula, coronavirus, global, macrophages, MERS, SARS, Saudi Arabia, therapeutic, vaccine, WHO

## Abstract

The first case of Middle East respiratory syndrome coronavirus (MERS-CoV) was identified in the year 2012, which spread rapidly and increased to more than 2200 in 2018. This highly pathogenic virus with high mortality rate is among one of the major public health concerns. Saudi Arabia remains to be the most affected region with the majority of MERS-CoV cases, and currently, no effective drugs and vaccines are available for prevention and treatment. A large amount of information is now available regarding the virus, its structure, route of transmission and its pathophysiology. Therefore, this review summarizes the current understanding of MERS-CoV's pathogenesis, treatment options and recent scientific advancements in vaccine and other therapeutic developments, and the major steps taken for MERS prevention control.

Middle East respiratory syndrome coronavirus (MERS-CoV) was identified as a zoonotic virus, whose mode of transmission is from animals to humans. The origin of the virus is believed to be bats, from which it was then transferred to camels. Camels are currently regarded as a major host for MERS-CoV. It has also been identified as the most significant source for human infections [1]. The isolates of MERS-CoV from camels and humans have also been found to more than 99% identical [2]. While camels have been identified as the primary source of MERS-CoV infection through both indirect as well as direct contact, the specific route and role of camels in disease transmission is yet to be identified.

The pathogenic agent of Middle East respiratory syndrome is a new coronavirus which was initially identified from the respiratory content of a patient who was infected, and died, as a result of infection from a mysterious viral disease showing pneumonia like symptoms in Saudi Arabia in 2012 [3]. Initially, a group of healthcare personnel working in a hospital in Jordan contracted a respiratory infection in April 2012, the source of which was not known [4]. Later in June 2012, an elderly businessman with severe pneumonia associated with kidney failure was admitted to a Saudi hospital. The coronavirus detected from his sputum was not known before and for a while it was referred to as human Coronavirus Erasmus Medical Center (hCoV-EMC) [5]. Following that case, another patient from the Middle East with severe respiratory infection, who was transferred to the UK, was found to be infected with the same virus. On analyzing and comparing the samples from the Jordan outbreak with the latter ones, the same virus hCoV-EMC was recognized as causing this new and severe form of respiratory infection. It was later titled as MERS-CoV [6]. Since its first detection to this date, this virus has spread in countries across Middle East and Europe [7]. The highest number of cases has been identified in Saudi Arabia. Infections in European countries have been brought in by travel from the Middle East [7,8].

From 2012 through the end of December 2018, the number of confirmed cases of MERS-CoV globally reported to the WHO was 2279 with 806 associated deaths, which corresponds to a fatality rate of approximately 35.36%. Saudi Arabia was on the top of the list of countries with 1901 reports of confirmed cases, including 732 related deaths with a fatality rate of approximately 39% [9]. Due to its high mortality rate (∼36%) [9,10] and pathogenicity, nonavailability of vaccine or any other definite treatment, MERS-CoV is considered a major challenge to global health and presents a pressing need for the research and development of definite therapeutic options and adequate management to prevent its infection [11,12].

The interest in coronaviruses was reignited after the 2002 outbreak of Severe Acute Respiratory Syndrome Coronavirus (SARS-CoV) and 2012 outbreak of MERS-CoV. Researchers have tried to understand pandemic potential of MERS-CoV by studying its emergence and ecology. Studies are being carrying out to assess how this virus causes disease so that novel therapeutics and vaccines can be developed. The mode of transmission of MERS-CoV is yet to be established, but it is assumed to have come from bats [13]. Camels found in the Arabian Peninsula may serve as intermediate hosts for human infection [14].

This review will focus on studies trying to elucidate mechanisms by which MERS-CoV escapes host-immune system and causes disease. We will also examine the recent advances in the development of novel therapeutics and vaccines.

Figure 1

**Figure 1.**
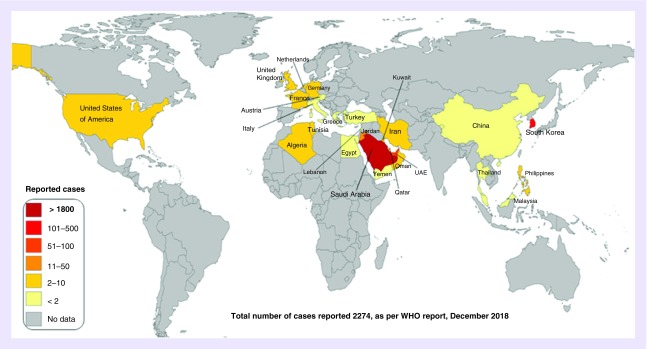
**Global map of confirmed MERS-CoV infections, 2012–2018.** Figure modified from [15].

## Characteristics of coronavirus & major proteins

Coronaviruses are positive stranded RNA viruses, and while most of these infect animals, particularly bats, a minor number can also cause human diseases [16]. Human coronaviruses can be broadly divided into types, α and β coronavirus [17]. MERS-CoV is from the β-coronavirus family [18]. It has four major surface proteins that help the virus to enter the cells *viz.* envelope protein (E), spike (S) protein, nucleocapsid (N) protein and membrane (M) protein. The spike (S) protein is a transmembrane glycoprotein made up of S1 and S2 subunits. The S protein is crucial for virus entry through binding and fusion to host cells. The S1 subunit has a receptor binding domain (RBD) that binds with the DPP4 receptor of the host [18,19]. The S2 subunit contains heptad repeats H1 and H2 which forms the main membrane fusion unit [20]. The E protein is required for assembly, intracellular transport and budding of virus [21]. The M protein has its role in viral morphogenesis and assembly [22]. N proteins and S, E and M proteins interact to form complete virus particles [23]. In addition to these structural proteins, MERS-CoV has two large polyproteins called pp1a and pp1ab. These proteins are broken down by proteases to form various nonstructural essential proteins, such as enzymes [24,25]. Recent studies have revealed that these viral structural and nonstructural proteins can be exploited as novel targets for therapeutic purposes [26–28].

## Clinical presentation

The infection caused by MERS-CoV has an average incubation time of 5 days (2–14 days range). The host, in this time period, shows no symptoms of infection. The clinical symptoms of the disease range from mild symptoms of upper respiratory infection like cough, fever and myalgia to severe forms such as pneumonitis, as well as respiratory failure. Patients may also suffer from abdominal pain, appetite loss, nausea, diarrhea, vomiting and other gastrointestinal symptoms. The less commonly occurring symptoms include hemoptysis and diarrhea without any hint of fever [29]. Studies advocate that chronic illnesses, such as chronic heart disease, kidney disease, diabetes and hypertension, increase the risk of MERS-CoV infection and its severity [30], though further proof is required.

MERS-CoV can alter antigen presentation, host immune response and modulate the apoptotic pathways and mitogen-activated protein kinase pathways [7].

## Pathogenesis

Before the discovery of MERS-CoV, SARS-CoV was considered the most pathogenic coronavirus. However, the higher pathogenicity of MERS-CoV was apparent by the higher number of deaths caused by this virus. Similar to the virus of SARS, MERS-CoV infects and replicates in the human airway epithelial cells and suppresses the production of interferons [31,32]. However, unlike SARS-CoV, the MERS virus exhibits wider tissue tropism [33,34]. MERS-CoV can also induce pro-inflammatory cytokines but lacks in production of innate antiviral cytokines compared with SARS-CoV. Suggesting MERS-CoV induces delayed pro-inflammatory response and attenuates innate immunity, which suggests that MERS-CoV is more lethal compared with SARS-CoV [35–37]. Primarily the MERS virus interacts with the host DPP4 receptor through its spike (S) protein after entering the respiratory tract. DPP4 receptors are present on the epithelial surface of various human organs such as, the lungs, kidneys, liver, bone marrow, thymus and intestines [38]. The systemic distribution of DPP4 facilitates the dissemination of virus in the human body. Expression of DPP4 on the respiratory tract is mainly on type I and type II pneumocytes, endothelial cells, nonciliated bronchial epithelial cells and a few forms of hematopoietic cells [39,40]. The abundance of the receptor DPP4 is greater on the epithelial cells lining the lower airways and alveoli and lesser on the epithelial surface of upper conducting airways and nasal cavity [41]. Recent findings have suggested that a prior existing pulmonary ailment might increase the chances of such individuals contracting MERS, as chronic pulmonary diseases results in enhanced DPP4 expression [40].

CoV *nsp1* is a serious virulence factor, which facilitates the biological actions of MERS-CoV. Studying the *nsp1* can advance our understanding of pathogenicity of MERS-CoV and facilitate development of better therapeutics. Host gene expression in infected cells is suppressed by MERS-CoV *nsp1*, which also promotes virus assembly or budding in *in vitro*, leading to efficient virus replication, suggesting *nsp1* is also critical for MERS-CoV replication and promotes production of virus particles in the host [42]. The severity of MERS-CoV infection is relatively more in patients with co-morbid conditions, such as chronic lung disease, renal failure, diabetes and others with compromised immune systems [9].

The understanding of MERS-CoV pathogenesis has been limited due to nonavailability of patient autopsy or pathological samples from the patients. Our understanding of the disease pathogenesis is based entirely on *in vitro* studies. Studies were conducted on animal models and human lung cell lines, and the account from a single autopsy [43]. *In vitro* studies revealed that MERS-CoV can easily replicate in several cultured human cells, as well as, in the differentiated and nondifferentiated human epithelial cells [34,44]. The antigens of MERS-CoV were found on the macrophages in the alveoli of the infected human lung explants, ciliated and nonciliated bronchial epithelial cells, endothelial cells and pneumocytes [33,45]. These findings were in sync with the findings made in the single autopsy where MERS-CoV antigen was detected on pneumocytes, endothelial cells and epithelial cells of the airways and few on macrophages [43,45].

Macrophages are the important phagocytic cells of innate immune system which help remove the pathogenic substances from the body and present their antigens to the T cells. The cytokines as well as chemokines produced by the macrophages help in destroying the pathogens, adjusting the immune system and maintaining tissue homoeostasis [46]. However, in MERS, the virus-infected macrophages contribute considerably to the development of disease symptoms [47]. The infection with MERS-CoV of human epithelial cells induces the release of pro-inflammatory chemokines and cytokines from the monocyte-derived macrophages. It is believed that these chemokines/cytokines cause inflammatory changes and tissue injury through infiltration of immune cells in the lower respiratory tract [47]. The patients suffering from MERS clinically manifest pneumonia, which is progressive in nature and has a large number of macrophages and neutrophils found in the fluids present in the lungs [3,48]. Studies conducted on rhesus macaques have shown the lung tissue infiltration of macrophages and neutrophils on MERS-CoV infection, though their respiratory symptoms were milder as compared with humans [49]. Many scientists believe that this sequestration of immune cells contribute to the development of lymphopenia seen in patients with MERS. The progress in the severity of pneumonia and respiratory dysfunction in the MERS patients is also attributed to cytokine/chemokine induction [30,50]. Zhou *et al*. found that MERS-CoV can efficiently replicate inside macrophages and, hence, can overcome the host immune system. [47]. Thus, these phagocytes act like reservoirs and means of transportation for these viruses, helping to replicate and disseminate, such as the HIV virus [51].

Infection of epithelial cells with the MERS virus induce slow, but significant, IFN type I and II responses [36]. Macrophages release pro-inflammatory chemokines and cytokines such as IL-1β, IL-6 and IL-8 upon MERS-CoV infection [37]. Similarly, MERS-CoV infection in blood monocyte-derived macrophages and dendritic cells leads to the release of chemokines and cytokines, for example, IL-2, IL-3, CCL-2, CCL-3 and RANTES [37,48]. Infection of activated T cells induces apoptosis via different pathways which may also explain the occurrence of lymphopenia [52]. With the present available knowledge, it is difficult to describe the exact pathogenesis of MERS-CoV, but it seems that viral replication in the macrophages results in extreme cytotoxicity and triggers the induction of pro-inflammatory chemicals which may lead to MERS-associated complications.

A small number of epithelial, pneumocytes, lymphoid aggregates and inflammatory cells of submucosal glands were positive for MERS spike protein in nonhuman primates (NHPs). Whereas, MERS spike antigen were positive in epithelial layers of submucosal bronchial glands, lungs of NHPs and in some cells in BALTs [53]. Dual Immunohistochemistry method was applied using monoclonal antibody against MERS spike protein and CD26, the staining showed that MERS was found in CD26 positive cells but were negative in NHPs [53].

Two NHPs, the common marmoset and rhesus macaque model were established for MERS-CoV infection by three different research groups [49,54–59]. The clinical symptoms included respiratory disease which was mild in nature and could be diagnosed with radio imaging and computed tomography [54,56,59] and respiratory disease of severe nature showing fatal clinical symptoms requiring euthanasia [54,59]. Exposure to the MERS-0 infectious clone (icMERS-0) strain in Rhesus monkeys is reported to cause respiratory disease, wherein virus antigen has been detected. Respiratory disease in such cases was found to be transient and mild in nature, which resolved by 30 days from infection. Pulmonary disease was also found to be mild upon MERS-CoV infection in NHPs. Earlier studies found ocular, intratracheal or intranasal exposure of MERS-EMC isolate to cause lethal disease. However, it has been argued that lethality was due to manipulations of marmoset, which have higher sensitivity than macaque species [54,57]. Cases of mild to moderate symptoms even after higher viral titer have also been reported in other studies [56]. Small animals, such as hamsters and mice, have been found to be resistant to MERS-CoV infection and development of models of severe respiratory disease has been a challenge [60].

## Therapeutics against MERS-CoV infection

The identification of highly pathogenic MERS-CoV suggests that coronaviruses present an incessant and long-term hazard to humans. Development of effective therapeutic and prophylactic agents to contain their infections is an urgent need, and yet currently no antiviral treatments against MERS-CoV are available. MERS-CoV is known to interact with host cell surface receptor DPP4 or CD26 with its spike S protein, which subsequently leads to its entry in the host cell [61]. Our knowledge regarding the exact mechanisms that follow thereafter is still limited and requires more research. Due to lack of definite treatment, supportive therapy remains the only solution. Present development consists of the previous experiences of other coronavirus diseases like SARS-CoV, which have been studied in *in vitro* and *in vivo* models. Tremendous efforts are being made in this direction and various antiviral and related therapies have been identified. Use of humanized monoclonal antibodies, convalescent plasma and therapeutic peptides has been shown efficacious. Repurposing of the currently available drugs is also being tried to extend their efficacy against MERS-CoV. Some potential options are discussed in detail below.

## Repurposing of drugs

The concept of using clinically available drugs to treat new diseases is known as repurposing. In this method, a new target profile is created for the existing drugs through screening of large molecular databases. Owing to advances in computational approaches and development of effective antiviral agents, repurposing has become faster. Using high-throughput screening, researchers have been able to screen large libraries of drugs against novel targets and evaluate their antiviral activity *in vitro* [62,63]. Some of repurposed drugs have shown potent antiviral activity against MERS-CoV, for example, ribavirin, nitazoxanide and hexachloropene [64]. Some other studies have repurposed the drugs by using a combination of two or more existing drugs. Wilde *et al*. combined alisporivir with ribavirin for enhanced antiviral activity against MERS and SARs-CoV [65]. In another study on MERS-CoV infected marmosets, hybrid of ritonavir/lopinavir and IFN-β1b had positive effect [66].

## Convalescent plasma

Convalescent plasma therapy utilizes plasma or whole blood from people who have been infected with viral diseases and recovered. This therapy has been used during outbreaks when no particular medicines or vaccines are available for treatment [67]. The use of convalescent plasma has been indicated to be an efficient therapeutic strategy for diseases like MERS-CoV [68]. Studies have been carried out to confirm its feasibility and safety in treating MERS; however, the data are insufficient. There are a few drawbacks of this therapy, as often large-scale screening is required to obtain sufficient amount of antibodies from potential donors [69].

## Monoclonal & polyclonal antibodies

Monoclonal antibodies have been commonly used in the diagnosis of various diseases. Therapeutics based on monoclonal antibodies have been used successfully in the therapy of various diseases [70,71]. The potential of this approach was acknowledged against coronaviruses during the SARS outbreak [72,73]. And when the other deadly coronavirus attacked humans, this previous knowledge helped greatly in the improving the response against the risk of MERS-CoV.

Initial studies suggested that MERS-CoV RBD domain in S1 glycoprotein represents a suitable target for the development of neutralizing monoclonal antibodies [62,74–76]. Hence, the idea to develop neutralizing mouse mAbs as a strategy to prevent the entry of MERS-CoV into host cells was led by Du *et al*. [75]. mAbs were made by vaccinating mice with IgG1 Fc to which recombinant MERS-CoV S1 was fused [75]. Consequently, Mersmab1, the most effective murine mAb was developed that targeted MERS-CoV RBD, and successfully neutralized MERS-CoV infection in Vero E6, Huh-7 and Calu-3 cells [76]. These studies indicated the potential of humanized mAbs as efficient curative agents against infection caused by MERS-CoV. Around April 2014, three independent studies first reported to have developed complete humanized MERS-CoV neutralizing mAbs [77,78]. All these humanized mAbs specifically targeted the MERS-CoV RBD glycoprotein. The efficacy of human mAbs against MERS-CoV infection was first exhibited by Qiu *et al*. [79]. They were able to completely treat MERS-CoV infection of lethal nature with a single dose of humanized mAbs in the hDPP4 transgenic mouse [79].

Development of humanized monoclonal antibodies against emerging viral diseases requires a significant production cost and poses as a major drawback. To overcome this obstacle and to reduce the cost, extremely powerful neutralizing mAbs which can be given in smaller doses without compromising the efficacy is needed. One strategy to achieve this could be to design humanized mAbs with exceptional binding attraction and targeting them against the potential targets, such as RBD.

## Peptides as potential antiviral therapeutic agents

Recently, interest has been generated in the development of peptide therapeutics as potential drug targets for different pathogens. Currently, more than 140 peptide therapeutics are lined up for clinical evaluation presenting peptide research as a component of pharmaceutical research [80]. As compared with chemical drugs, peptide drugs show high specificity toward the target as well as little side effect and drug tolerance [19]. Many peptide therapeutics have shown positive results against viral infections, for instance RVFV-6 peptide against rift valley fever virus and Kn2-7 peptide derived from scorpion venom against HIV-1 [81–83]. Antimicrobial peptides are cited as potential novel antiviral therapeutics against coronaviruses [84]. Antimicrobial peptides are produced by host immune system upon initial exposure to pathogens. These are gene encoded and positively charged peptides that are selectively toxic against their targets [85,86]. This selectivity is due to their positive charge which attacks the negatively charged membrane bilayer of microbes [87]. The antimicrobial peptides block the receptors present on the host cell's surface, which in turn inhibit different steps of viral fusion and replication causing virolysis and activation of host's adaptive immune response [88].

## Future perspective

Since the emergence of MERS-CoV, research has greatly enhanced our knowledge of the pathogenesis caused by it and other contemporary coronavirus, such as SARS-CoV. The efforts toward development of vaccines against this deadly virus have also been continuously increasing leading to the emergence of promising interventions. The two viruses, in other words, SARS and MERS have some common challenges in the development of an efficacious vaccine. As evident from reports, the aged population is more vulnerable to MERS-CoV. The lesson learnt from preclinical studies of SARS-CoV suggests that vaccines fail to protect aged animals, while being effective in young ones. Similarly, in clinical settings, the risk of mortality is even higher in individuals with chronic conditions or an immunocompromised state. An effective vaccine, thus, should offer universal protection, including the vulnerable populations. To do so, there is a need to evaluate the promising vaccines in the comorbid chronic conditions and the immunocompromised rodent models. Since the virus can comfortably replicate in macrophages, the risk of vaccine-derived immunopathology cannot be negated and must be considered using heterologous challenge models. Further, balance must be struck between protection and excessive immune activation while evaluating a successful vaccine candidate. The vaccine development against MERS-CoV is mostly influenced by the SARS-CoV. However, to aid the development of better vaccines, there is need to further enhance the knowledge of pathology of MERS-CoV and outline critical differences between SARS and MERS-CoV. mAbs offers a window of opportunity, as a few candidates have shown potency in *in vitro* testing. However, care needs to be taken in humanizing mAbs so as to minimize the antimouse antibody response. Epitopes identified from mouse neutralizing mAbs can be neutralized for humanizing MERS-CoV. mAbs targeting RBD are reported to have higher potency than therapies directed against other S protein regions of MERS-CoV, as these could recognize critical residues for DPP4 binding. However, changes in such critical residues may render these mAbs ineffective and lead to development of escape mutant strains of the virus. As discussed, a few promising peptides have also been developed against MERS-CoV. The antiviral activity, stability and solubility of these peptides can be further improved similar to the peptides developed against HIV. This will lead to the development of optimized next generation peptides having better inhibitory action directed against MERS-CoV. Alternatively, as a novel approach, the peptide inhibitors can be combined with mAbs (e.g., RBD specific). The combination can be evaluated for the synergistic effect against divergent and resistant strains. Studies are also needed to explore novel delivery technologies and optimizing vaccine immunity with a suitable combination of adjuvants. In summary, there have been encouraging results in the area of the development of MERS-CoV vaccines in preclinical settings. However, there are challenges related to efficacy, safety and drug delivery that need further consideration before proceeding to clinical trials. Focused research in this direction can help reduce the disease burden caused by MERS-CoV and prevent outbreaks, especially among the aged and immunocompromised population.
